# Use of Natural Spoken Language With Automated Mapping of Self-reported Food Intake to Food Composition Data for Low-Burden Real-time Dietary Assessment: Method Comparison Study

**DOI:** 10.2196/26988

**Published:** 2021-12-06

**Authors:** Salima Taylor, Mandy Korpusik, Sai Das, Cheryl Gilhooly, Ryan Simpson, James Glass, Susan Roberts

**Affiliations:** 1 Jean Mayer United States Department of Agriculture Human Nutrition Research Center on Aging Tufts University Boston, MA United States; 2 Computer Science and Artificial Intelligence Laboratory Massachusetts Institute of Technology Cambridge, MA United States; 3 Friedman School of Nutrition Science and Policy Tufts University Boston, MA United States

**Keywords:** energy intake, macronutrient intakes, 24-hour recall, machine learning, convolutional neural networks, nutrition, diet, app, natural language processing

## Abstract

**Background:**

Self-monitoring food intake is a cornerstone of national recommendations for health, but existing apps for this purpose are burdensome for users and researchers, which limits use.

**Objective:**

We developed and pilot tested a new app (COCO Nutritionist) that combines speech understanding technology with technologies for mapping foods to appropriate food composition codes in national databases, for lower-burden and automated nutritional analysis of self-reported dietary intake.

**Methods:**

COCO was compared with the multiple-pass, interviewer-administered 24-hour recall method for assessment of energy intake. COCO was used for 5 consecutive days, and 24-hour dietary recalls were obtained for two of the days. Participants were 35 women and men with a mean age of 28 (range 20-58) years and mean BMI of 24 (range 17-48) kg/m^2^.

**Results:**

There was no significant difference in energy intake between values obtained by COCO and 24-hour recall for days when both methods were used (mean 2092, SD 1044 kcal versus mean 2030, SD 687 kcal, *P*=.70). There were also no significant differences between the methods for percent of energy from protein, carbohydrate, and fat (*P*=.27-.89), and no trend in energy intake obtained with COCO over the entire 5-day study period (*P*=.19).

**Conclusions:**

This first demonstration of a dietary assessment method using natural spoken language to map reported foods to food composition codes demonstrates a promising new approach to automate assessments of dietary intake.

## Introduction

National recommendations encourage self-monitoring of energy intake to support healthy weight management [1-3]. Mobile phone–based apps are widespread and include features like food imaging and portion guides [4-6]. However, none of the available apps appear to be more accurate or decrease user burden compared with earlier methods [7], and high user burden combined with limited accuracy are major barriers to routine use. Moreover, high user burden is associated with modifications to usual eating habits that result in atypical energy and nutrient intakes during the measurement period [8].

The use of speech understanding technology for the assessment of food intake is in its infancy but has tremendous potential to reduce the burden of dietary assessment and increase method accuracy, in particular when combined with technologies to map reported foods to their appropriate food composition code for automated nutritional analysis. We have developed an app called COCO (Conversational Calorie Counter) Nutritionist [9], which combines natural spoken language with machine learning to enhance the capture of self-reported dietary intake and map it to food codes in widely used food databases. The primary goal of this study was to conduct a feasibility study evaluating COCO for measurement of energy intake compared to a recommended method [10].

## Methods

### Overview

This paper describes the first evaluation of COCO for automated self-reported dietary intake. The evaluation was conducted by nutrition research staff at the Jean Mayer USDA Human Nutrition Research Center on Aging at Tufts University in Boston, Massachusetts, with data collection and analysis implemented by individuals who had no role in the development of COCO.

### COCO Nutritionist

COCO was developed primarily by two members of the research team (JG and MK) in collaboration with the nutrition researchers (SBR, SKD, and CG). A detailed description of the development of the app is provided in Multimedia Appendix 1. Briefly, COCO uses machine learning, specifically deep convolutional neural networks (CNN) [9], to accomplish two tasks: (1) tag each word in a user’s natural language description of an eating event as a specific food/drink, with its quantity and brand name and/or description [11]; and (2) map each food item to a matching entity in one of several national food databases [12]. The databases used were the United States Department of Agriculture standard reference and branded foods [13], the University of Minnesota database [14], foods scraped from restaurant websites, and the Nutritionix database [15]. A front-end iOS app [16] makes calls to a back-end Python Flask [17] server that runs the trained CNN models.

Food database measuring units (eg, cup) and amount units (eg, 1, 2) are ranked using string matching. For example, if the user says “cup,” it will rank the unit “cup” as the top match. If there are no exact matches, then it defaults to the unit that the user logged for that food item most often previously; if the user never ate that food before, then it defaults to the most popular unit logged by all users. Each user’s food logs are stored in a PostgreSQL [18] database on Amazon Web Services [19]. Thus, a user’s subsequent logs are customized such that the paired food codes and amounts of user-described foods they have eaten previously are ranked higher or at the top. The input is a spoken or written meal description (eg, “For dinner, I had a bowl of chili with cheddar cheese”). The output is a list of top-15 matching database foods per tagged food item (ranked according to the dot product similarity scores between a vector representing the user's description of the food they ate and a vector representing each food database entry), along with their ranked quantities (ranked according to keyword matching), predicted number amount (eg, 1, if the user logged “a cup”), and corresponding nutrition facts from the selected nutrition database, calculated for each food item. If there are duplicates for foods in different databases, the current system will return both, ranking based on matching the wording in the database versus the user’s language.

Participants implicitly confirmed the top-1 food code and food amount proposed by COCO, by not making any changes, or they picked a more suitable food code from the database if the top food code was not the right match, by scrolling through a list of 15 options. If none of the presented options were a good match, participants could delete the food and repeat. A similar process was used for food amounts.

### Evaluation of Participants and Informed Consent

#### Participants and Informed Consent

The research protocol for evaluation of the new COCO app was reviewed and approved by the Tufts Health Sciences Institutional Review Board, and informed consent was obtained from each participant prior to enrollment. In total, 35 participants were recruited using flyers, word of mouth, and emails to a mailing list of individuals requesting to be contacted about nutrition research study opportunities or expressing interest in the app. To be eligible, participants had to be at least 18 years old and generally in good health. Individuals were ineligible if they did not have an iPhone with iOS 10.2 or higher, were unwilling to log their food intake for 5 consecutive days and complete 2 diet recalls, or had a graduate degree in nutrition. Enrollment occurred between June 2018 and June 2019.

#### Evaluation Protocol

We developed an evaluation protocol for the COCO prototype that was consistent with recommendations for development of new food intake assessment tools [20]. Participants were enrolled in a 5-day study, and completed all components remotely. They were provided with a written overview of the study, including instructions on how to download and use the COCO app, as well as a short video providing tips on use. Participants were asked to use COCO to record the amounts and types of all consumed food and drinks for 5 consecutive days. The recording of intake could be completed using natural spoken language utterances or manual text entry. If a day of recording was missed, they were asked to log an additional day to complete a total of 5 days of entries. A member of the nutrition team (ST) called participants to schedule 2 calls for the 24-hour food recalls (one per day) on day 3 and later. The diet recalls were rescheduled if the previous day’s food logging was missed. Each 24-hour dietary recall took approximately an hour to complete. Following completion of both diet recalls, participants were instructed to complete food logging for the remainder of the study.

The diet recalls used the recommended multiple-pass interviewer-administered method [10], and started with an uninterrupted listing by the participant of all foods and beverages consumed the previous day. In addition, the interviewer went through a forgotten foods list, querying the subject on categories of foods that have been documented as frequently forgotten, followed by a final probe listing all the food items consumed, confirming details of the food and serving sizes. A standard food amount booklet [21] was used to assist in portion size estimation of consumed foods. It has eight sections: (1) the forgotten foods list, (2) glasses and mugs, (3) bowls, (4) mounds, (5) circles, (6) grid and thickness blocks, (7) wedges, and (8) shapes and chicken pieces. The 24-hour diet recalls were analyzed using Food Processor (version 10.13.1; ESHA Research Inc).

Demographic questions were also completed by participants, including for age, sex, race, ethnicity, education, weight, and height. A study satisfaction survey on the use of the app was sent to participants to complete at the end of the study, and a US $20 gift card was mailed to their home address.

### Analysis

Data from COCO on energy and macronutrient intakes by study day, amounts of information collected via text versus voice data capture, and percentage of food codes proposed by COCO and revised by participants were captured by the Massachusetts Institute of Technology development team without access to the 24-hour dietary recall data, and provided to the Tufts team for statistical comparison of the methods.

Data for the two days when both methods were performed were averaged for each method. Reported mean energy intakes and percent of energy from protein, fat, carbohydrate, and alcohol were compared between the two methods using paired samples *t* tests and Pearson correlations. In addition, for the COCO data obtained over 5 days, the time-distributed effects of energy consumption were evaluated in relation to the sequence of recalls and the day of the week using multiple pairwise comparisons of means with a Bonferroni correction. For comparisons across the number of recalls, we also used multiple comparisons of means, as well as simple linear regression with and without adjusting for weekday versus weekend effects, to examine time series trends in average energy consumption by the numbered recall performed.

All tests were performed for biologically plausible observations, defined a priori as 2-day average energy consumptions <5000 kcals (1 participant was excluded for implausibility). Analyses were performed using STATA (release 15; StataCorp LLC). The significance level was set at α≤.05.

## Results

Participants were 25 females and 9 males with a mean age of 28 (range 20-58) years, and mean BMI of 24 (range 17-48) kg/m^2^ (Table S1 in Multimedia Appendix 1). When comparing the 2-day averages of COCO and 24-hour recall, we found no significant difference in energy intake (mean 2092, SD 1044 versus mean 2030, SD 687 kcal/d; *P*=.70) or percent energy from protein (mean 16, SD 3 versus mean 17, SD 5; *P*=.54), fat (mean 35, SD 9 versus mean 36, SD 11; *P*=.27), or carbohydrate (mean 50, SD 11 versus mean 50, SD 9; *P*=.89) between the 2-day comparison of COCO and the 24-hour recall method (Table 1).

**Table 1:** Energy and macronutrient intakes and number of eating events reported with COCO Nutritionist and 24-hour recall (mean of the same two days for each method).^a^

Variables	24-hour recall (N=34), mean (SD)	COCO Nutritionist (N=34), mean (SD)	*P* value
Energy intake, kcal/day	2030 (687)	2092 (1044)	.70
Percent energy from protein	17 (5)	16 (3)	.54
Percent energy from fat	36 (11)	35 (9)	.27
Percent energy from carbohydrate	50 (9)	50 (11)	.89
Number of meals and snacks	4 (1)	4 (1)	.37

^a^Paired *t* tests were used to compare energy and macronutrient intakes across methods.

Mean values were very similar between the methods, as was the number of reported eating events (meals and snacks). On average, there were 4 items in the 24-hour recall that were not reported in COCO (components of composite food items and beverages), and 2 items in COCO that were not reported in the 24-hour recall (snacks, candy, and beverages), reflecting in part the different ways that composite foods (eg, a hamburger, consisting of meat, bread, and other items) were processed by the two methods. There were also significant Pearson associations between 2-day averages of COCO and 24-hour recalls for percent energy from protein (ρ=0.66; *P*<.001), carbohydrate (ρ=0.58; *P*<.01), and fat (ρ=0.38; *P*=.03), with *R*^2^ values of 0.119-0.438 (Figure S1 in Multimedia Appendix 1).

**Figure 1:**
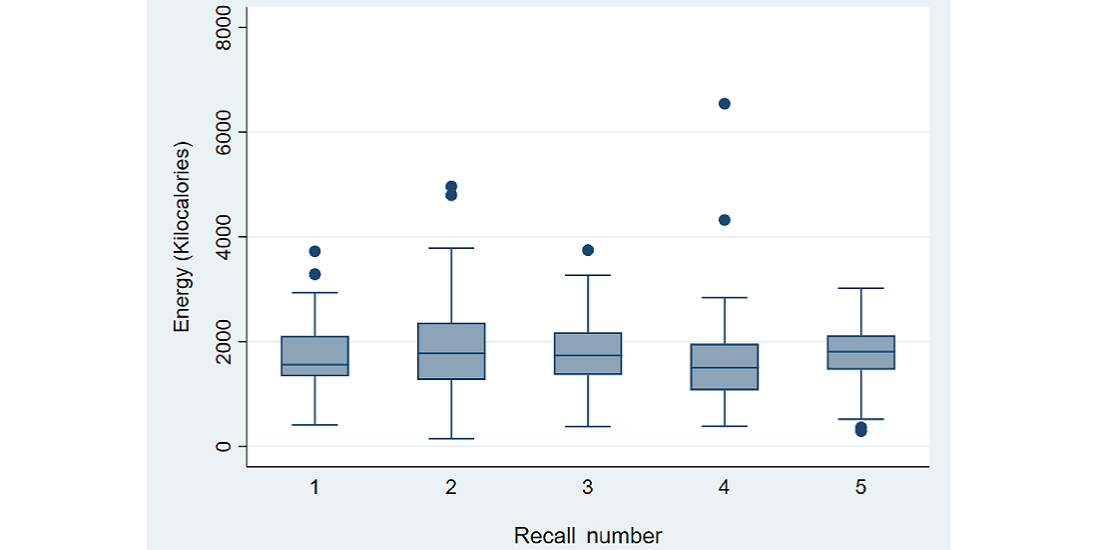
Energy intake reported over 5 consecutive days through COCO Nutritionist (n=34). There was no significant trend in energy intake reporting (*P*=.19).

As shown in Figure 1, there was no significant trend over time in reporting of total energy by COCO (*P*=.69), which remained insignificant when controlling for weekend versus weekday effects (*P*=.73). We additionally evaluated how participants entered data, and the accuracy of mapping foods to appropriate food codes. Most foods were logged by typing rather than by speaking (Table 2). Similar ratios of spoken and written descriptions contained mentions of brands or preparation technique, and the percentage was relatively low at ~15%. Spoken meal descriptions were more likely to mention the units of foods (36.1% versus 24.4%). Furthermore, because the editing function of COCO allowed for participant revision of the food type, brand, or description and unit amount (ie, 1, 2, or 3 cups), how often participants revised the COCO-suggested option was also tracked (8.1% of the time for food name/description/brand and 27.9% of the time for unit amount).

**Table 2:** Comparison of the food entities logged with spoken versus written natural language in COCO Nutritionist.^a^

Variables	Spoken	Written
Mean number of foods logged per person per day	1.38	7.06
Total of all logged foods, %	28.4	71.6
Mean percentage of foods with specified brands/preparation method	15.6	15.0
Foods with quantities, %	30.7	22.1
Food code identified correctly in default option, %	100	99.9
Food measuring unit (eg, cup) identified correctly in default option, %	83.5	84.1
Food amount unit (eg, 1, 2) identified correctly in default option, %	74.5	84.7

^a^Food code, units, and amounts measured correctly at first try are defined as the user not changing the default.

## Discussion

### Principal Findings

This study demonstrates that an app using natural spoken language to capture participant self-reports can be combined with automatic mapping of identified foods to information on food composition in national databases to generate estimates of mean self-reported energy and macronutrient intakes—and of the number of meals and snacks eaten—that are not significantly different from those obtained with the gold-standard multiple-pass, interviewer-administered 24-hour recall method. In addition, the lack of any negative trend in reported energy intake by the new COCO app across the 5-day study period was a positive indication that participants were able to complete their reporting without undue burden. To our knowledge, this is the first report of using natural spoken language to collect dietary data in a process that allows for mapping of collected information to food codes to support automated nutrient analyses. Although the data are preliminary, they suggest that this new approach may facilitate accurate, low-cost, and lower-burden methods for dietary tracking for health and weight management.

### Comparison With Prior Work

A crucial aspect of this work was demonstration that data collected with natural spoken language can be automatically mapped to a suitable food code in national food databases, something that has not been done previously and allows for automated and instantaneous calculation of dietary intake. This novel feature of COCO will reduce the cost and user burden of dietary assessment, and distinguishes it from previous work using natural spoken language that had a dietician assign food codes to each consumed food [22]. Our demonstration of a relatively low percentage of revisions in the default mapping of collected information to food codes and portion sizes suggests that systems combining voice recognition and writing allow for flexibility in participant use, which may in turn enhance acceptance and sustainable use.

### Limitations

Limitations of the study include a relatively small number of features in the COCO app, compared to fully developed apps that include food photography. In addition, the comparator method was self-reported dietary intake by 24-hour recall, which is considered a gold standard for dietary reporting but nevertheless may not reflect absolute dietary intakes. The population sample was relatively small in this first study, and one participant was excluded from data analyses for implausible reporting. Additional work is needed to further refine COCO and to compare collected data with dietary intake of known origin.

Concerning future improvements in COCO focused on making the system as helpful and low-burden as possible, a next step would be to implement a more sophisticated chatbot that can provide personalized food recommendations [23]. For example, the agent could respond if the user asks a question such as “How many calories are in a cup of milk?” or “How many grams of fat have I eaten today?” and recommend foods based on which nutrients the user is missing and their diet preferences. If the user is low in iron, the system could recommend, for example, spinach or steak (both foods high in iron), depending on prior food choices. In addition, taking photos of meals, which is simpler for some users than verbal logging, could provide complementary information to that provided by natural language. Multitask learning research has shown that neural networks trained on multiple input sources and prediction tasks (eg, one side may take an input image, while the other takes an input sentence) have improved performance on both modalities over using each separately [24]. Diet apps use computer vision already; however, to our knowledge, no one has combined language understanding with computer vision.

### Summary

Self-monitoring food intake is a cornerstone recommendation in lifestyle interventions for weight management. However, self-monitoring food intake is burdensome with current apps, and frequently inaccurate. This first demonstration of using natural spoken language to map reports of food intake to food composition codes in national food databases and generate automatic assessments of dietary intake demonstrates a promising new approach to substantially reducing user and investigator burden in assessment of dietary intake while supporting the accuracy of reported data.

## Appendix

Multimedia Appendix 1 Supplementary tables, figures, and methods.

## Abbreviations

CNN: convolutional neural network
COCO: Conversational Calorie Counter
USDA: United States Department of Agriculture
