# Supplementary material for: Use of Natural Spoken Language With Automated Mapping of Self-reported Food Intake to Food Composition Data for Low-Burden Real-time Dietary Assessment: Method Comparison Study
Source: J Med Internet Res. 2021 Dec 6;23(12):e26988. doi: 10.2196/26988 (PMC8691405; doi:10.2196/26988)
Supplement: Multimedia Appendix 1 [file jmir_v23i12e26988_app1.docx]

**Supplement**

Table S1…………………………………………….……………………….Page 2

Figure S1…………………………………………………………...……….Page 3

Supplementary Methods …………………………………………..……….Page 4

**Table S1. Study Population**

|  | Mean | Range |
| --- | --- | --- |
| N=26 females + 9 males |  |  |
| Age, years | 28 | 20-58 |
| Height, in | 66 | 60-75 |
| Weight, lb | 147 | 99-279 |
| BMI, kg/m2 | 24 | 17-48 |

**Figure S1: Comparison of percent of energy from protein, fat and carbohydrate for dietary data collected over the same two 24h periods using 24-h recall and COCO.**


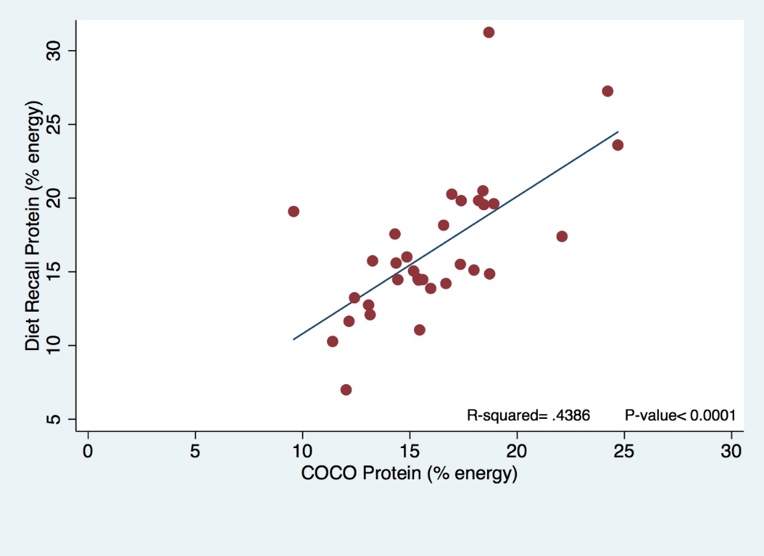


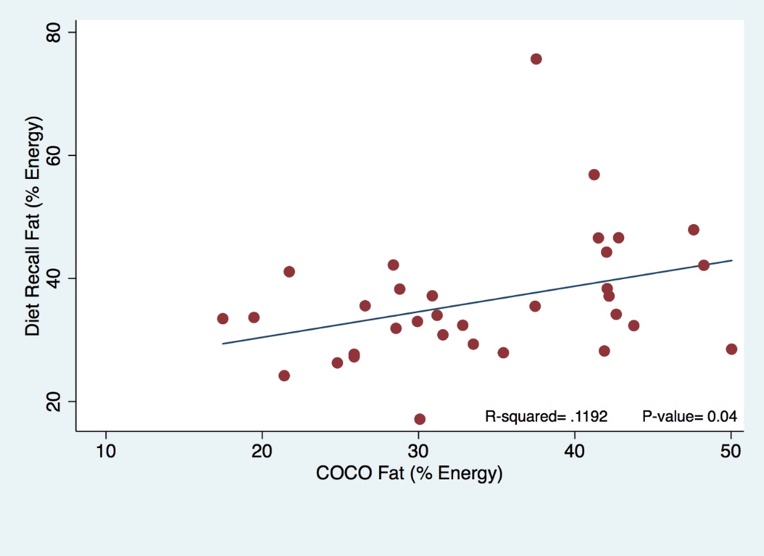


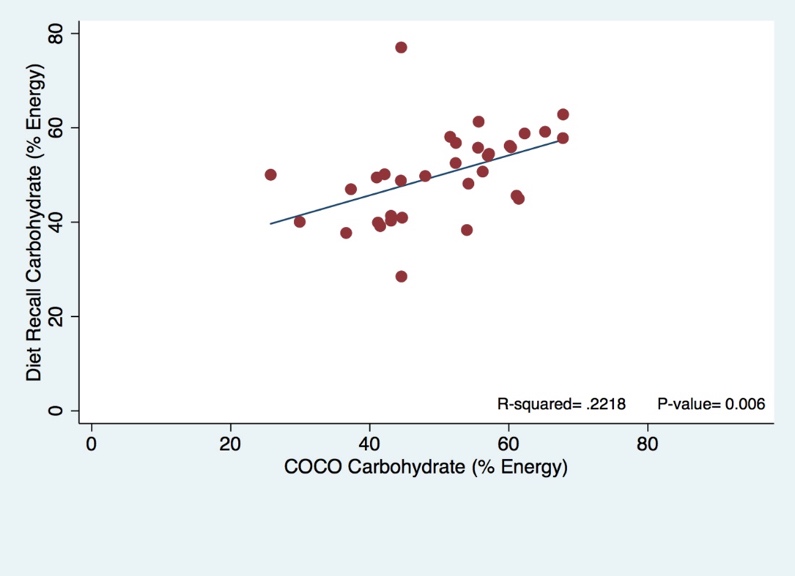


**Supplementary Methods: Development of *COCO Nutritionist***

*
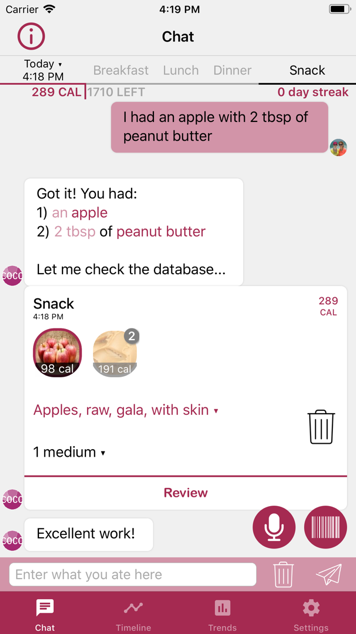
*Due to the aforementioned benefits of a natural spoken language interface for nutrition and other domains, as well as the prevalence of smartphones in the United States, we have both deployed a back-end server and developed a front-end iOS prototype for our spoken diet tracking system (see *Coco Nutritionist* in the Apple store). The user interface, displayed in Fig. 1, illustrates the “chat” panel in which users log their meal either with speech (i.e., via the microphone icon in the bottom-right corner), or by typing.

Our innovation allows users to more easily interact with a dialogue system by speaking *naturally*. Typically, conventional dialogue systems rely heavily on manual feature engineering and a set of heuristics for mapping from user queries to database entries. There is a fundamental mismatch between how people describe objects, and how the matching entities are represented in a structured database. For example, a person may say they had a slice of “toast,” rather than a piece of bread. However, the USDA food database may only contain “bread, toasted,” which is not an exact word match, as well as incorrect matches, such as French toast and toasted nuts. Historically, researchers dealt with this mismatch through text normalization (e.g., removing punctuation, lowercasing). To avoid this pipeline of text regularization and word matching lookup, we have designed a novel deep learning model that is fed raw input and handles the text mismatch internally. Rather than requiring manually defined semantic dictionaries of synonyms, or checking for plurals and misspellings, our novel approach *automatically* identifies text segments that are *semantically similar* (Korpusik, 2017c; Korpusik, 2018).

*Fig. 1: The user interface for the spoken diet tracking system prototype, Coco Nutritionist. In this scenario, the user logged “I had an apple with 2 tbsp of peanut butter” by tapping the microphone icon and speaking naturally.*

**Database Mapping with Semantic Vectors**

The natural language understanding component of a diet tracking system requires mapping a spoken or written meal description to the corresponding USDA food database matches. Following the work of (Korpusik, 2019a), the first step consists of a convolutional neural network (CNN) followed by re-ranking to generate a ranked list of the top-n USDA foods (n=500 in our experiments) per tagged food segment. We employed two steps to achieve a system that directly selects the best USDA matches for a given meal description: 1) we constructed a CNN model that learns vector representations for USDA food items through a binary verification task (i.e., whether or not a USDA item is mentioned in a meal description), and 2) we computed dot products over learned embeddings to rank the food database entries.

*Learning Semantic Embeddings with CNNs*

As shown in Fig. 3a, our model is composed of a shared 64-dimension embedding layer, followed by one convolution layer above the embedded meal description, and max-pooling over the embedded USDA food name. The text is tokenized using spaCy.^[[1]](#footnote-1)^ The meal CNN computes a 1D convolution of 64 filters spanning a window of three tokens with a rectified linear unit (ReLU) activation. During training, both the USDA input's max-pooling and the CNN's convolution over the meal description are followed by dropout (Srivastava, 2014) of probability 0.1,^[[2]](#footnote-2)^ and batch normalization (Ioffe, 2015) to maintain a mean near zero and a standard deviation close to one. A dot product is performed between the max-pooled 64-dimension USDA vector and each 64-dimension CNN output of the meal description. Mean-pooling^[[3]](#footnote-3)^ across these dot products yields a single scalar value, followed by a sigmoid layer for final prediction.^[[4]](#footnote-4)^ This design is motivated by our goal to compare the similarity of specific words in a meal description to each USDA food.

To prepare the data for training, we padded each text input to 100 tokens,^[[5]](#footnote-5)^ and limited the vocabulary to the most frequent 3,000 words, setting the rest to UNK. We trained the model to predict each (USDA food, meal) input pair as a match or not (1 or 0) with a threshold of 0.5 on the output. The model was optimized with Adam (Kingma, 2014) on binary cross-entropy loss, norm clipping at 0.1, a learning rate of 0.001, early stopping after the loss stops decreasing for the second time on the validation data (i.e., 20% of the data), and mini-batches of 16 samples. We removed capitalization and commas from the USDA foods.

*Semantic Tagging and Re-ranking at Test Time*

At test time, rather than feeding the entire meal description into the CNN, we instead first perform semantic tagging as a pre-processing step to identify individual food segments (Korpusik, 2014; Korpusik, 2016), and subsequently rank database foods via dot products with their learned embeddings. A CNN tagger (Korpusik, 2017a; Korpusik, 2019b) labels tokens as Begin-Food, Inside-Food, Quantity, and Other. Then, we feed a food segment into the pre-trained embedding layer to generate vectors for each token. Finally, we average the vectors for tokens in each tagged food segment (i.e., consecutive tokens labeled Begin-Food and Inside-Food), and compute the dot products between these food segments and each previously computed and stored USDA food vector.^[[6]](#footnote-6)^ The dot products are used to rank the USDA foods in two steps: a fast-pass ranking, followed by fine-grained re-ranking that weights important words more heavily. For example, simple ranking would yield generic milk as the top hit for 2% milk, whereas re-ranking focuses on the property 2% and correctly identifies 2% milk as the top match.

- **Ranking:** initial ranking of USDA foods using dot products between USDA vectors and food segment vectors.
- **Re-ranking:** fine-grained word-by-word cosine similarity^[[7]](#footnote-7)^ ranking of the top-30 hits with weighted distance $D$.

$$D= \sum_{i} \alpha_{i} {max}_{j}(w_{i}\cdot w_{j})+ \frac{1}{N}\sum_{j} \beta_{j} {max}_{i}(w_{i}\cdot w_{j})$$

where $N$ refers to the length of the tagged food segment. The left-hand term finds the most similar meal description token $w_{j}$ to each USDA token $w_{i}$, weighted by the probability$\alpha_{i}$ that token was used to describe the USDA food item in the training data. In the same way, the right-hand term finds the most similar USDA token $w_{i}$ to each meal token $w_{j}$, weighted by the probability $\beta_{j}$ that token $w_{j}$ was used to describe that USDA food item in the training data (see Fig. 2).^[[8]](#footnote-8)^


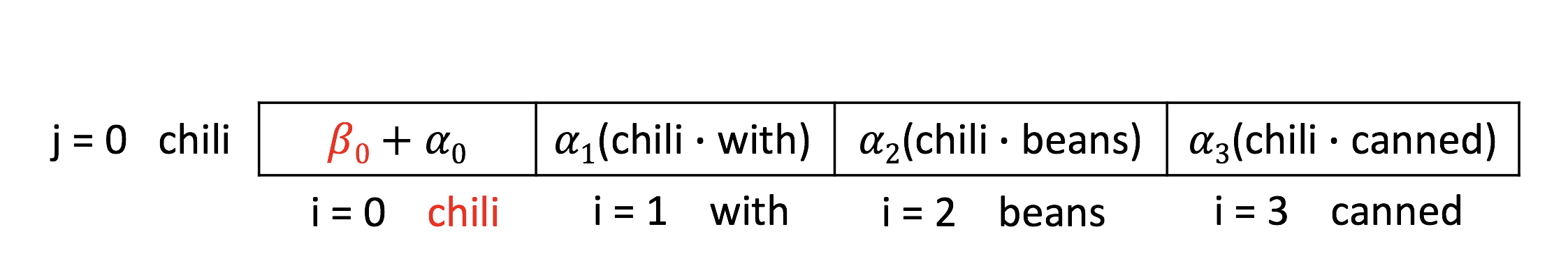


*Fig. 2: A reranking example for the food “chili” and matching USDA item “chili with beans canned.” There is only one* $\beta_{0}$ *term in the right-hand summation of the equation for* $D$*, since there is only a single token “chili” from the meal description.*

Note that the network was trained ***end-to-end****, without any pre-trained word embeddings or semantic tags*, and was not told where the food items were located in the meal description. Rather, the network had to *automatically infer* which words in the human-described meal corresponded to each USDA food, since the meals contained at least three foods, as well as non-food words. By successfully solving this task, the network learned meaningful vector representations of USDA food items, where foods from the same category lay close together in vector space (see Fig. 3b).

At test time, we ranked all the USDA foods by feeding a user's meal log through the CNN to generate a vector; we scored its similarity with each USDA food's learned vector. This yielded 64% top-5 food recall, significantly better than rule-based (Korpusik, 2017b).


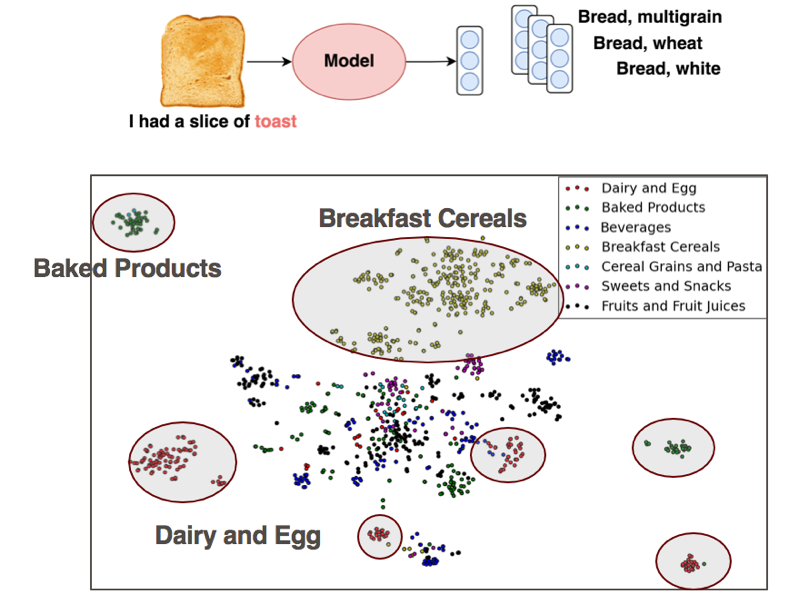

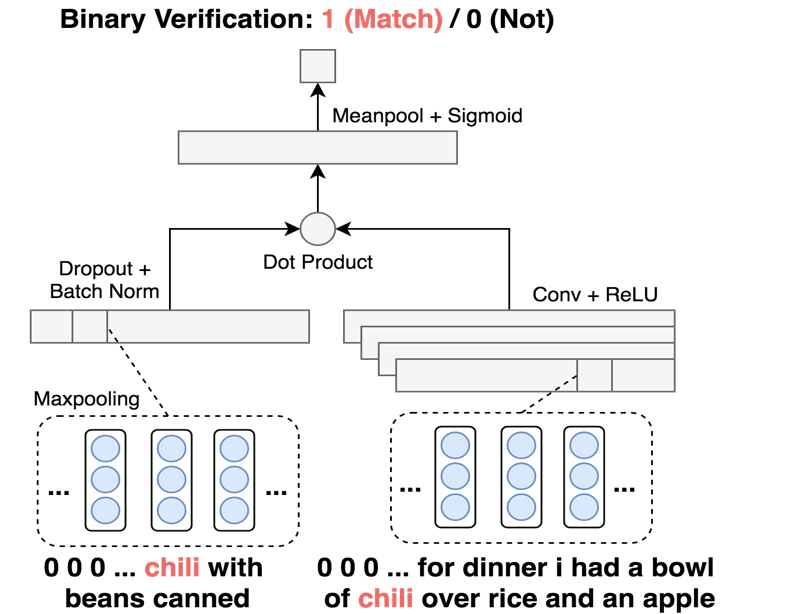

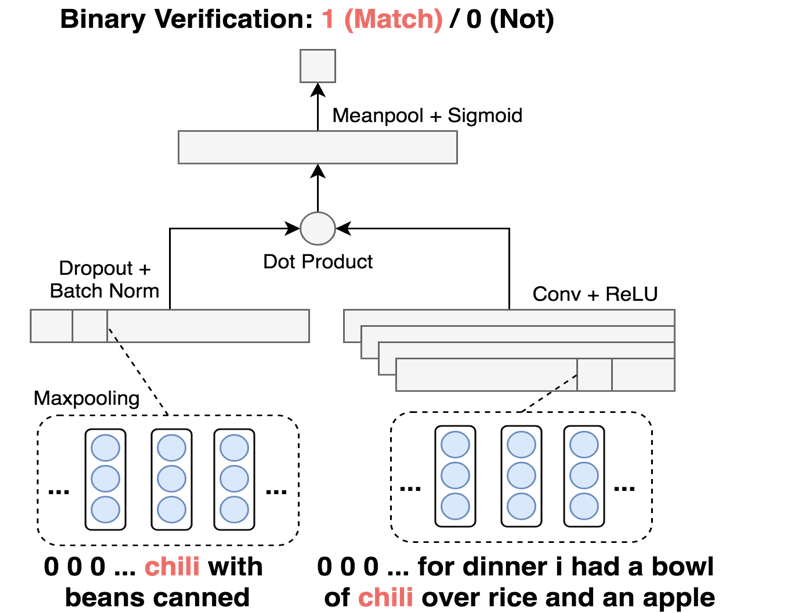

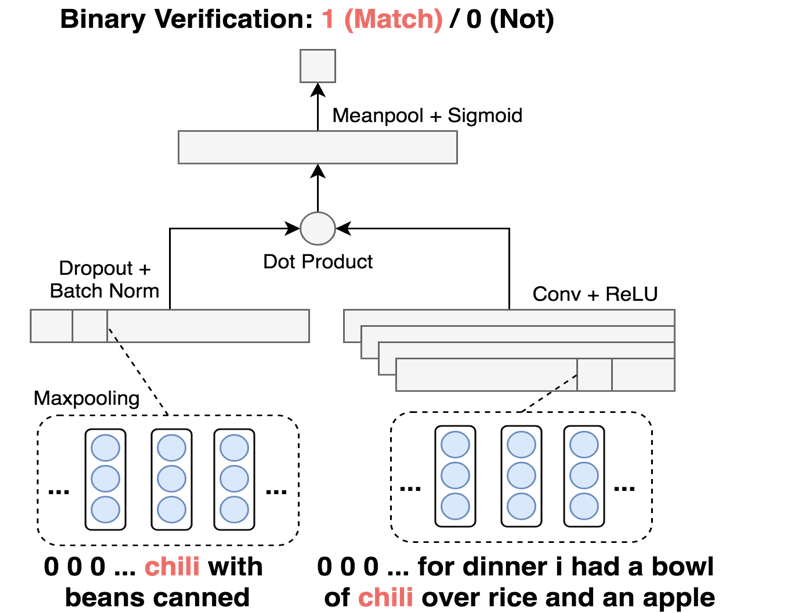

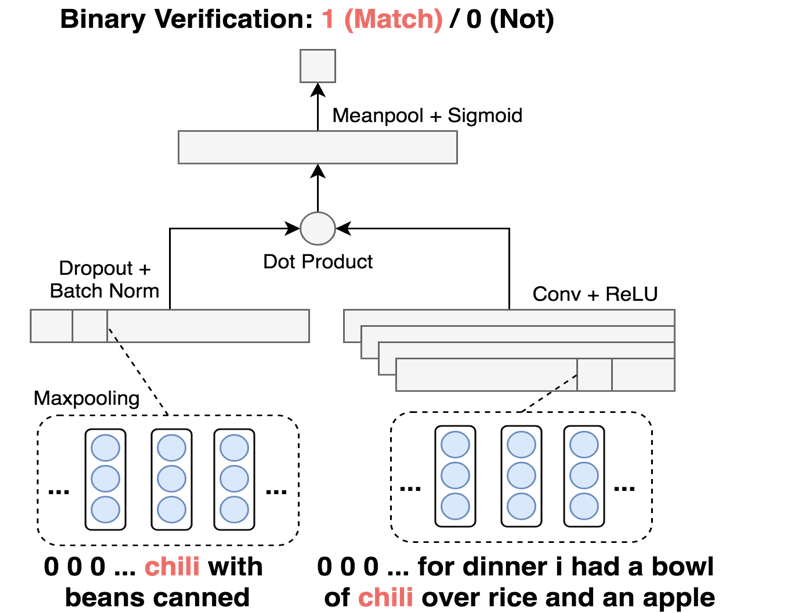

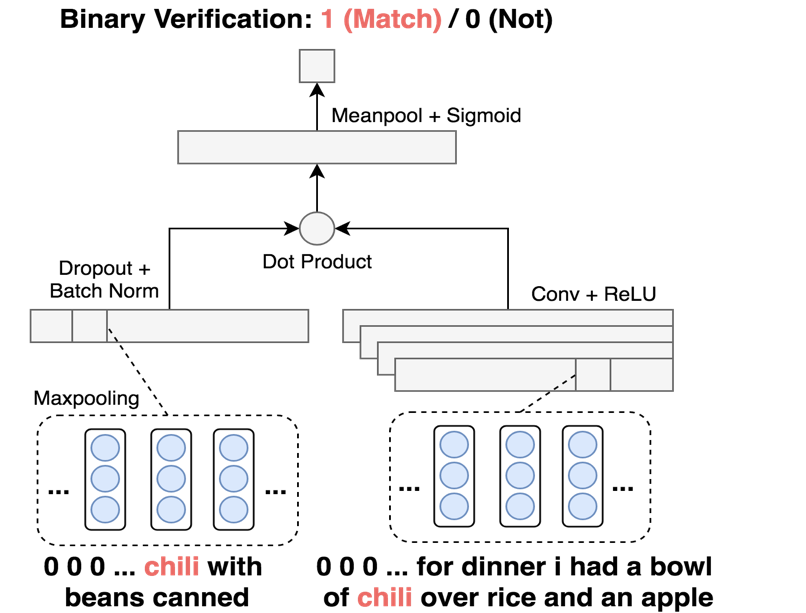

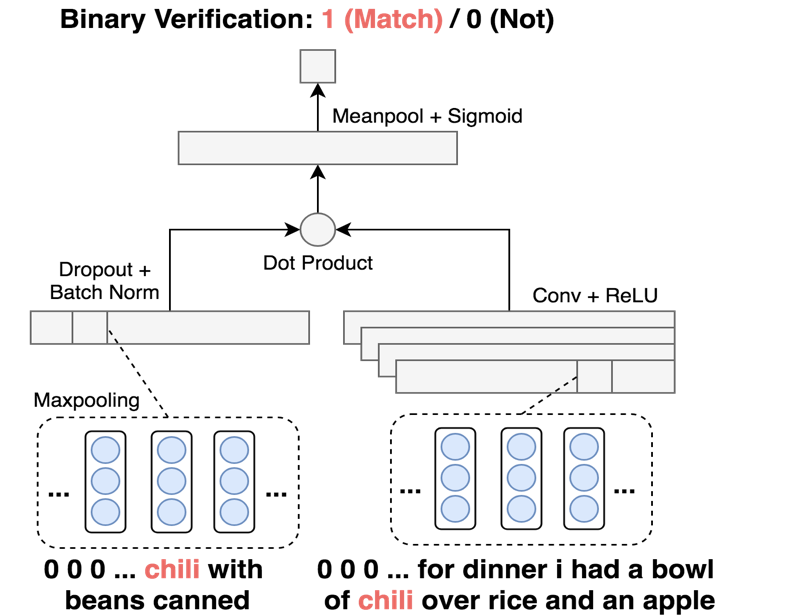

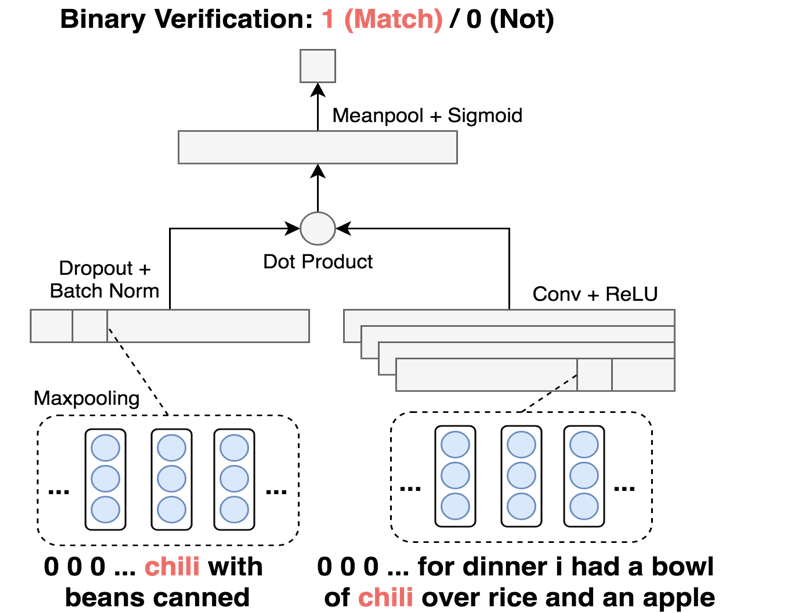

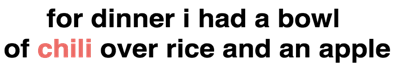

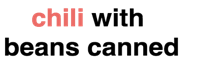


*Fig. 2: a) CNN for binary verification. b) Semantic embeddings of foods learned by the CNN.*

**Spoken Dialogue System Architecture**

The full system prototype (shown in Fig. 4), enables collection of real-world data for re-training the neural network models, as well as feedback from users interacting with the system. Initially, the user’s natural language meal description is sent from the client-side iOS application to a Python Flask server,^[[9]](#footnote-9)^ which is composed of the previously described CNN semantic tagger for predicting semantic tags (i.e., Begin-Food-Segment, Inside-Food-Segment, Begin-Quantity-Segment, Inside-Quantity-Segment, and Other) per token, and the database mapper composed of the two reranking steps previously described. In addition, a PostgreSQL database^[[10]](#footnote-10)^ stores the USDA foods and nutrition facts, the users’ usernames and unique IDs, as well as the meal logs. The client-side application is implemented in the Swift programming language,^[[11]](#footnote-11)^ and secure https calls are made to the server hosted on an Nginx proxy server^[[12]](#footnote-12)^ on a machine at MIT.

**System Evaluation**

In order to evaluate the system’s performance (i.e., top-1 and top-5 food and quantity recall over time), we launched an Amazon Mechanical Turk (AMT) crowdsourcing task (shown in Fig. 5. The Google recognizer transcribed the recordings. The stored transcripts and recordings will enable us to train a nutrition-specific speech recognizer. In total, 152,981 natural language meal descriptions were collected. By combining this speech data with the large number of written meal descriptions previously collected, we can leverage a powerful language model specific to the nutrition domain to guide our automatic speech recognition (ASR).


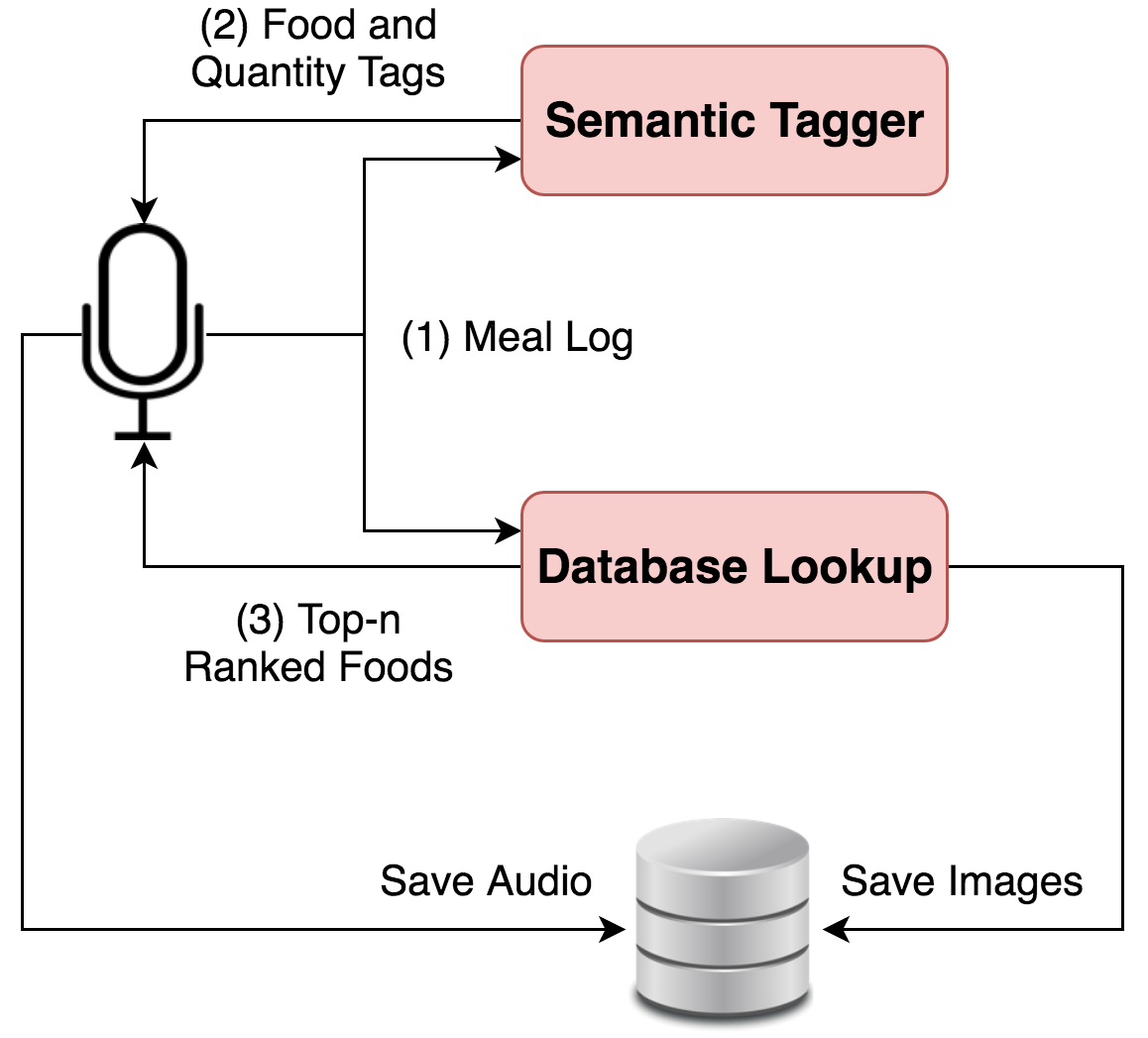


*Fig. 4: The full system architecture between the client (represented as a microphone) and the server (composed of the semantic tagger and database mapping via reranked embeddings).*

Workers were instructed to select the best matching foods and quantities from among the predicted top-n ranked results retrieved by the server, yielding an additional source of data with which to re-train the CNN models. The AMT tasks were released in batches of 1,000 over a period of several months, allowing for tracking of food and quantity recall over time. As shown in Fig. 6, recall improves over time since the ranked hits are sorted based on how often the food match was eaten. Therefore, as more workers interact with the system and select the correct food matches from the ranked results, the system learns from this data and improves over time. We also note that top-15 food recall plateaus at 100% recall, enabling us to reduce our top-n from 30 to only 15 matching foods. Finally, the top-5 quantity recall is consistently high, most likely due to individual foods often not containing more than five unit options.

Deploying the system prototype to the Apple Store provides another mechanism for evaluation. If we assume that the food selected by the user was indeed the correct match, then we may compute top-1 and top-5 recall scores for the real-world application as well. 16 months after launch to the Apple Store, *Coco Nutritionist* was downloaded onto over 30,000 iOS devices, with top-1 and top-5 food recall scores of 92.4% and 97.2%, respectively. The quantities and amounts are still using basic string matching algorithms, which we note have lower recall than the food prediction with the learned CNN embedding and reranking approach (i.e., only 87.2% top-1 quantity recall, and 85.9% for amount prediction).


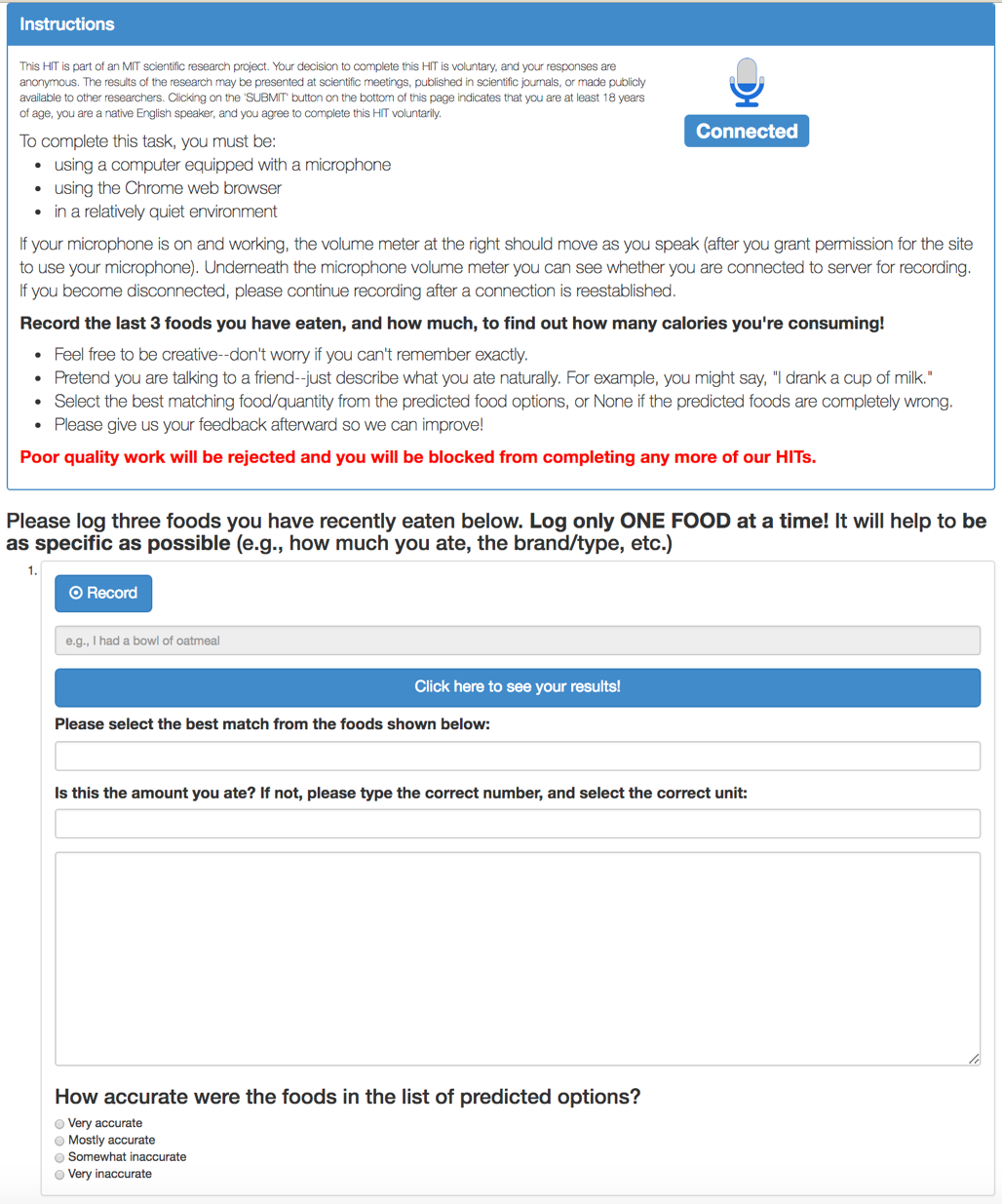


*Fig. 5: The Amazon Mechanical Turk task for system evaluation and ongoing data collection for re-training the neural network models. Workers were instructed to record three foods verbally, including quantities, and to select the correct food and quantity matches returned by the server.*

**Analysis of Spoken versus Written Natural Language**

An analysis of spoken versus written meal descriptions indicates that approximately 30% of the logs are spoken. Fig. 7 demonstrates that the distribution for speech has a longer tail, which indicates that speech is lengthier than writing. While most written logs tend to contain only one, two, or three words, the spoken logs’ probability mass is more spread out and highest for four words. Intuitively, people tend to be more descriptive and wordy when speaking because it is easier and more natural than typing, which is beneficial for a diet tracking application since increased specificity results in more accurate database mapping and, thus, higher food recall scores. In addition, the spoken meal descriptions contain fewer capitalized and punctuation characters than written (i.e., only 3.95% of spoken characters are uppercase, compared to 6.47% of typed characters, and only 0.37% of spoken characters are punctuation, whereas 1.42% of typed characters are punctuation), which we hypothesize is due to the more formal style of writing, while speech recognizers often omit punctuation.

**
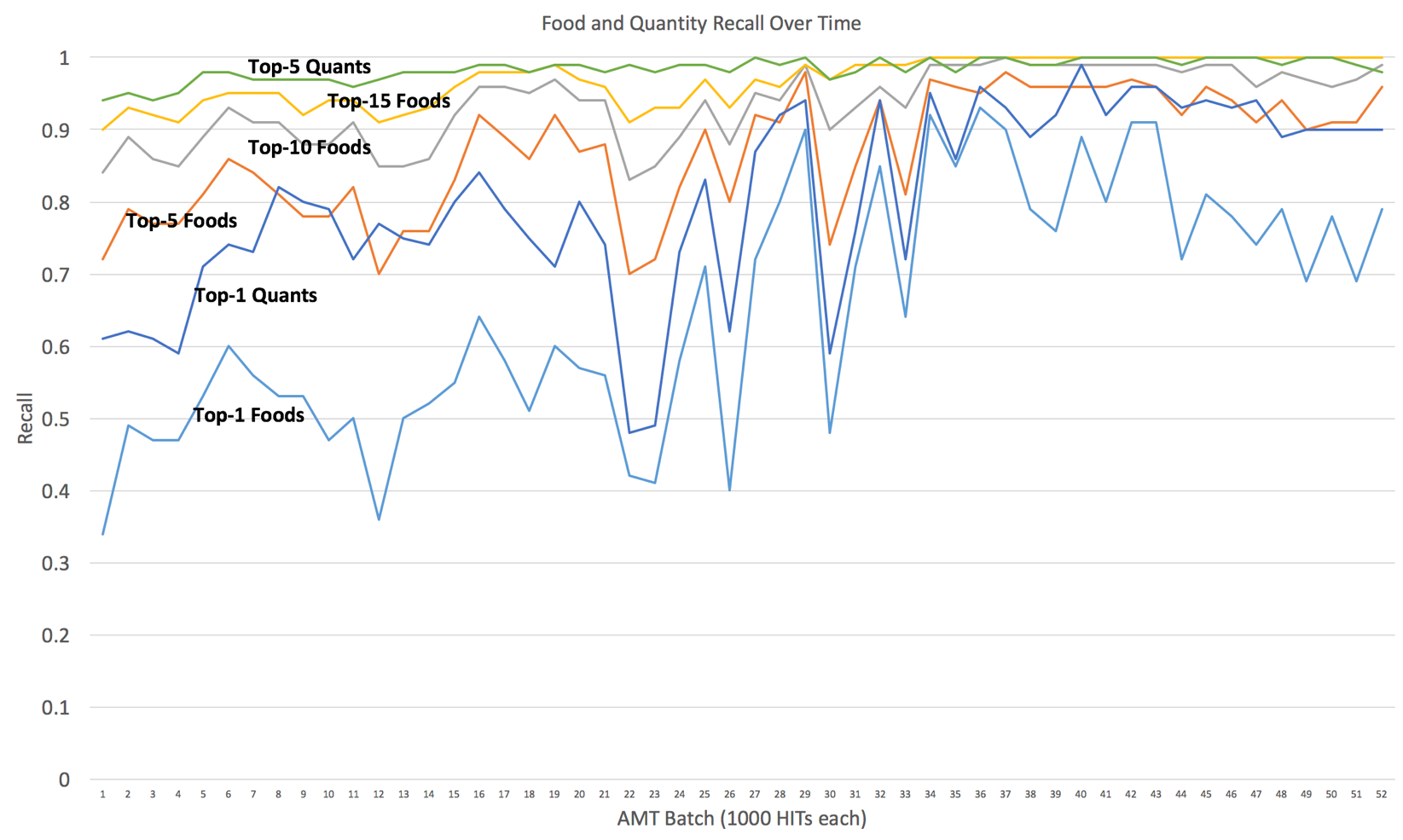
**

*Fig. 6: Food and quantity recall over time (i.e., per AMT batch of 1,000 tasks). The observed trend is that scores improve over time, as the system learns from interacting with more users.*


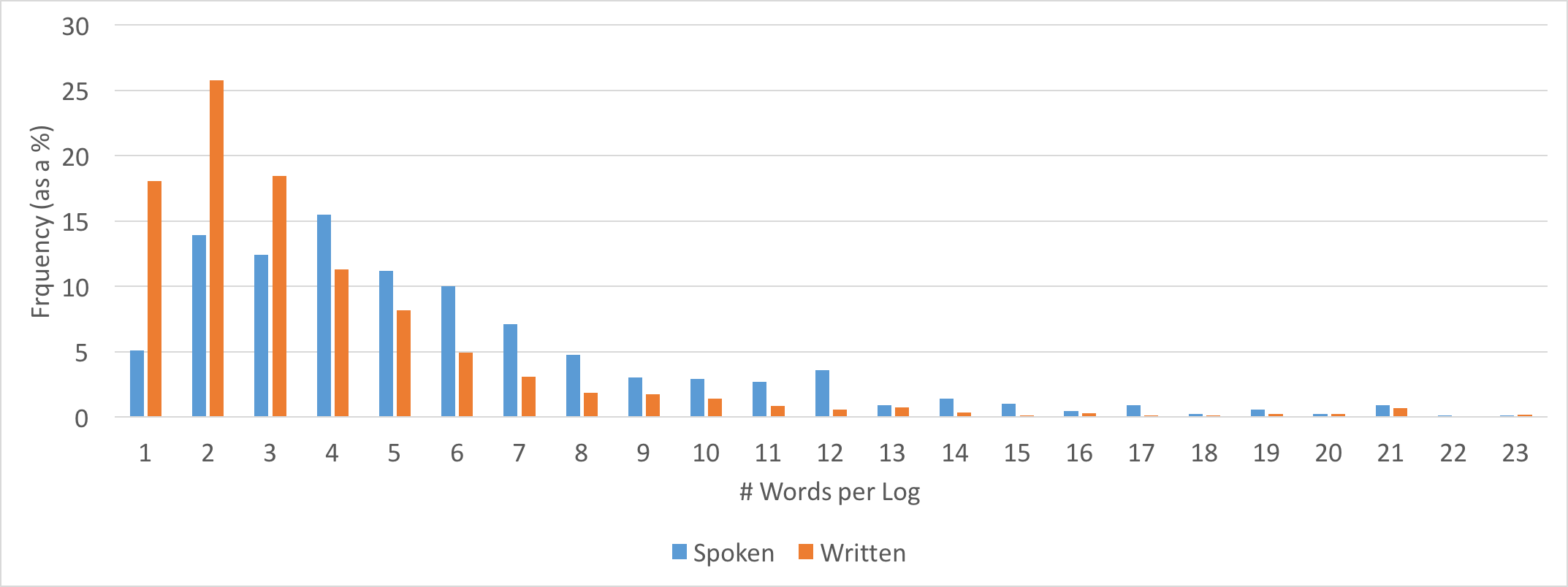


*Fig. 7: A histogram of the number of words per spoken or typed food log. The long tail in the spoken distribution indicates that users are more descriptive when speaking than typing, demonstrating that the speech modality is not only faster and easier, but also more accurate.*

**References**

Ioffe, S., & Szegedy, C. (2015). Batch normalization: Accelerating deep network training by reducing internal covariate shift. *arXiv preprint arXiv:1502.03167*.

Kingma, D. P., & Ba, J. (2014). Adam: A method for stochastic optimization. *arXiv preprint arXiv:1412.6980*.

Korpusik, M., Schmidt, N., Drexler, J., Cyphers, S., & Glass, J. (2014). Data collection and language understanding of food descriptions. In *2014 IEEE Spoken Language Technology Workshop (SLT)* (pp. 560-565). IEEE.

Korpusik, M., Huang, C., Price, M., & Glass, J. (2016). Distributional semantics for understanding spoken meal descriptions. In *2016 IEEE International Conference on Acoustics, Speech and Signal Processing (ICASSP)* (pp. 6070-6074). IEEE.

Korpusik, M., & Glass, J. (2017a). Spoken language understanding for a nutrition dialogue system. *IEEE/ACM Transactions on Audio, Speech, and Language Processing*, *25*(7), 1450-1461.

Korpusik, M., Collins, Z., & Glass, J. (2017b). Semantic mapping of natural language input to database entries via convolutional neural networks. In *2017 IEEE International Conference on Acoustics, Speech and Signal Processing (ICASSP)* (pp. 5685-5689). IEEE.

Korpusik, M., Collins, Z., & Glass, J. (2017c). Character-Based Embedding Models and Reranking Strategies for Understanding Natural Language Meal Descriptions. In *Interspeech*(pp. 3320-3324).

Korpusik, M., & Glass, J. (2018). Convolutional neural networks and multitask strategies for semantic mapping of natural language input to a structured database. In *2018 IEEE International Conference on Acoustics, Speech and Signal Processing (ICASSP)* (pp. 6174-6178). IEEE.

Korpusik, M., & Glass, J. (2019a). Deep Learning for Database Mapping and Asking Clarification Questions in Dialogue Systems. *IEEE/ACM Transactions on Audio, Speech, and Language Processing*.

Korpusik, M., Liu, Z., & Glass, J. (2019b). A Comparison of Deep Learning Methods for Language Understanding. *Proc. Interspeech 2019*, 849-853.

Mikolov, T., Sutskever, I., Chen, K., Corrado, G. S., & Dean, J. (2013). Distributed representations of words and phrases and their compositionality. In *Advances in neural information processing systems* (pp. 3111-3119).

Srivastava, N., Hinton, G., Krizhevsky, A., Sutskever, I., & Salakhutdinov, R. (2014). Dropout: a simple way to prevent neural networks from overfitting. *The journal of machine learning research*, *15*(1), 1929-1958.

1. https://spacy.io [↑](#footnote-ref-1)
2. Performance was better with 0.1 dropout than 0.2 or no dropout. [↑](#footnote-ref-2)
3. The inverse (mean-pooling before dot product) hurt performance. [↑](#footnote-ref-3)
4. Note that our approach would work for newly added database entries, since we can feed the new database food’s name into the pre-trained CNN to generate a learned embedding. This is the strength of using a binary prediction task, rather than a softmax output, so we do not have to re-train the network every time the database adds a new entry. [↑](#footnote-ref-4)
5. We selected 100 as an upper bound since the longest meal description in the data contained 93 words. [↑](#footnote-ref-5)
6. Our approach with CNN-learned embeddings significantly outperforms re-ranking with skipgram embeddings (Mikolov, 2013). For comparison, on breakfast descriptions, our model achieves 64.8% top-5 recall, whereas re-ranking with skipgrams only yields 3.0% top-5 recall. [↑](#footnote-ref-6)
7. Note that for the initial ranking, we use dot product similarity scores, but for word-by-word similarity re-ranking, we use cosine distance. These distances were selected empirically, where on the dataset of all foods, these metrics yielded higher food recall than using dot products for both ranking steps, or cosine distance for both, and Euclidean distance was the worst. [↑](#footnote-ref-7)
8. Although the sum appears biased toward longer USDA foods, the right-hand term is over each token in the food segment, which is fixed, and the left-hand term is normalized by the $\alpha$weights. Dividing $D$ by the number of tokens in the USDA food item hurt performance (39.8% recall on breakfast data versus 64.8% with the best model). [↑](#footnote-ref-8)
9. http://flask.pocoo.org/ [↑](#footnote-ref-9)
10. https://www.postgresql.org/ [↑](#footnote-ref-10)
11. https://swift.org/ [↑](#footnote-ref-11)
12. https://www.digitalocean.com/community/tutorials/how-to-secure-nginx-with-let-s-encrypt-on-ubuntu-16-04 [↑](#footnote-ref-12)
